# Sample purification and characterization of the α- and β-crustacyanin pigments from the American lobster for crystallographic and cryo-EM studies

**DOI:** 10.1107/S2053230X2600018X

**Published:** 2026-01-15

**Authors:** Maria Claudia Cedri, Harsh Bansia, Adolfo Amici, Thomas Collet, Paolo Moretti, Andrew A. McCarthy, Christoph Mueller-Dieckmann, Nadia Raffaelli, Tong Wang, Amedee des Georges, Michele Cianci

**Affiliations:** a Department of Agricultural, Food and Environmental Sciences, Università Politecnica delle Marche, Via Brecce Bianche, 60131Ancona, Italy; b CUNY Advanced Science Research Center City University of New York 85 Saint Nicholas Terrace New York NY10031 USA; c Department of Clinical Sciences, Università Politecnica delle Marche, Via Brecce Bianche, 60131Ancona, Italy; d Department of Life and Environmental Sciences, Università Politecnica delle Marche, Via Brecce Bianche, 60131Ancona, Italy; e European Molecular Biology Laboratory, EMBL Grenoble, 71 Avenue des Martyrs, CS-9018, 38000Grenoble, France; f Structural Biology Group European Synchrotron Radiation Facility (ESRF) 71 Avenue des Martyrs 38000Grenoble France; g Department of Chemistry and Biochemistry The City College of New York New York USA; h PhD Programs in Chemistry and Biochemistry, The Graduate Center City University of New York New York USA; Centro Nacional de Biotecnología – CSIC, Spain

**Keywords:** astaxanthin, crustacyanin, absorption, crystallography, cryo-EM, carotenoids

## Abstract

The striking blue hue of live *Homarus americanus*, known as the American lobster, arises from the interaction of astaxanthin with the carotenoprotein α-crustacyanin. The mechanism underlying the bathochromic shift of the chromophore from its unbound red form (λ_max_ = 472 nm) to the blue protein-bound form (λ_max_ = 631 nm) is the subject of attention from the food and nutraceutical industries for the development of versatile food colourants. Here, we present sample purification and characterization of the α- and β-crustacyanin pigments from the American lobster for crystallographic studies and cryo-EM. Moving from *H. gammarus* to *H. americanus* for complex isolation, together with an integrated biophysical characterization, resulted in the production, for the first time, of α-crustacyanin crystals that diffracted to 6.3 Å resolution at 〈*I*/σ(*I*)〉 = 1.0 and high-quality negative-stain and cryo-EM images.

## Abstract 2

The striking blue colour of the American lobster is caused by proteins that interact with the red pigment astaxanthin, causing its colour change. We have purified and characterized the pigments from the American lobster for crystallographic and cryo-EM studies.

## 1. Introduction

*Homarus americanus*, known as the American or Canadian lobster, or the Maine lobster due to its distribution throughout the Atlantic coast of North America, and the closely related *H. gammarus* or European lobster (Holthuis, 1991 ▸) are readily available from fishmongers and are widely recognized for their deep slate-blue hue when alive, while some may argue that their vivid colour change to bright red upon cooking garners even more attention.

The blue colouration of the lobster carapace is generated by the exocuticle carotenoproteins α-, β- and γ-crustacyanin (Cheesman *et al.*, 1966 ▸; Quarmby *et al.*, 1977 ▸). The α-crustacyanins from *H. americanus* and *H. gammarus* exhibit identical absorption spectra (λ_max_ = 631 nm) but behave differently in ion-exchange chromatography and polyacrylamide gel electrophoresis (Zagalsky & Tidmarsh, 1985 ▸). α-Crustacyanins are constituted of multiple copies of the β-crustacyanin heterodimer in which the chromophore, namely the carotenoid astaxanthin (3,3′-dihydroxy-β,β-carotene-4,4′-dione), is associated in a 1:1 stoichiometry with the protein subunits (Zagalsky, 1985 ▸). About 10–20% of the carapace carotenoprotein is present in another form, γ-crustacyanin, with λ_max_ = 625 nm, that differs from α-crustacyanin in molecular shape and charge but is identical in molecular weight and amino-acid composition (Zagalsky, 1985 ▸).

The absorption spectrum λ_max_ in the visible region of astaxanthin shifts drastically from λ_max_ = 472 nm in the unbound form, producing a visible red colour, to λ_max_ = 591 nm when bound in the β-crustacyanin heterodimer alone (Zagalsky & Cheesman, 1963 ▸; Cheesman *et al.*, 1966 ▸; Zagalsky & Tidmarsh, 1985 ▸), generating a blue-violet colour, and finally to λ_max_ = 631 nm, corresponding to the well known dark bluish colour of live lobster, when bound within β-crustacyanin in the α-crustacyanin complex.

The crystal structure of β-crustacyanin from *H. gammarus* has been resolved at 3.2 Å resolution (Cianci *et al.*, 2002 ▸) as a heterodimer formed by two types of apo lipocalin proteins (Grzyb *et al.*, 2006 ▸; Skerra, 2000 ▸) with molecular weights of 21 kDa (type I; CRTC; Cianci *et al.*, 2001 ▸; Habash *et al.*, 2004 ▸; Gordon *et al.*, 2001 ▸) and 19 kDa (type II; CRTA) and two noncovalently bound astaxanthin molecules (Cianci *et al.*, 2002 ▸). The structure of the blue-violet-coloured β-crustacyanin from *H. gammarus* elucidates the basis for the batho­chromic shift of the carotenoid spectrum to λ_max_ = 591 nm, identifying the coplanarization and polarization of astaxanthin as contributions to the bathochromic shift of astaxanthin (Cianci *et al.*, 2002 ▸).

**Figure 1:**
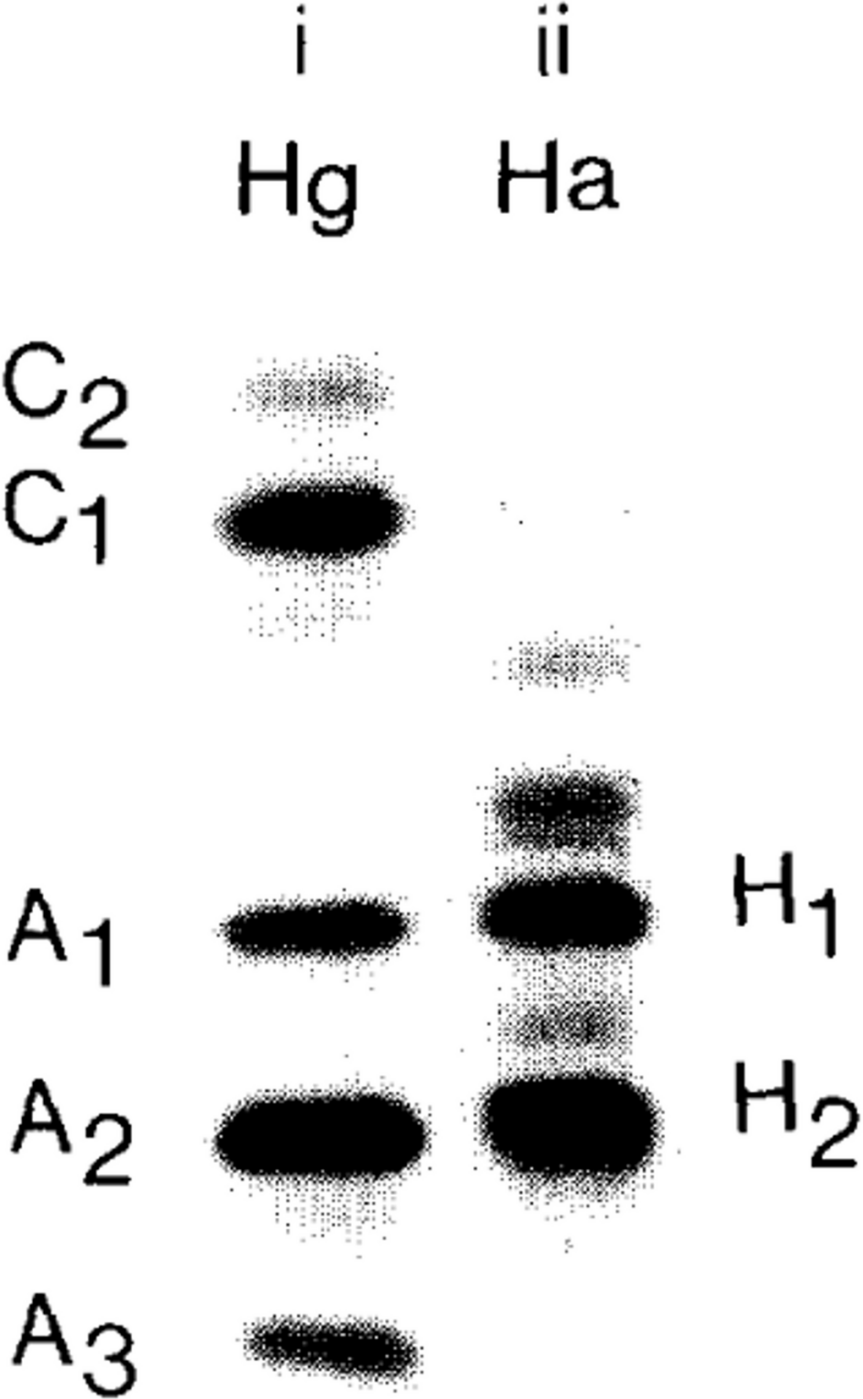
Comparison of the unit composition of *H. gammarus* and *H. americanus* α-crustacyanins. 6 *M* urea–PAGE of crustacyanin pigments from *H. gammarus* (left; Hg) and *H. americanus* (right; Ha). Reproduced with permission from Zagalsky & Tidmarsh (1985 ▸).

Previous investigations using small-angle X-ray scattering (SAXS; Dellisanti *et al.*, 2003 ▸; Chayen *et al.*, 2003 ▸) and negative-stain electron microscopy (Rhys *et al.*, 2011 ▸) of α-crustacyanin from *H. gammarus* derived various possible architectures of the complex up to a resolution of around 30 Å. Crustacyanin proteins from *H. americanus* are closely related to those from *H. gammarus* (Zagalsky & Tidmarsh, 1985 ▸). In the case of *H. gammarus*, five distinct subunits were evident on 6 *M* urea–PAGE gels, namely A1, C1 and C2 (type I, CRTC, 20 kDa) and A2 and A3 (type II, CRTA, 18 kDa), while for *H. americanus* only two major distinct subunits were evident, namely H1 (type I, CRTC) and H2 (type II, CRTA) (Fig. 1 ▸; Zagalsky & Tidmarsh, 1985 ▸; Ferrari *et al.*, 2012 ▸), thus suggesting a more homogeneous sample for structural investigations.

Here, we report sample preparation from α- and β-crustacyanin pigments purified from *ex vivo* material from *H. americanus* for X-ray crystallography and cryo-electron microscopy (cryo-EM) studies. α-Crustacyanin and β-crustacyanin complexes have been purified *ex vivo* from the *H. americanus* carapace together with γ-crustacyanin. Since γ-crustacyanin is a minor pigment in the lobster carapace, this study focused on β- and α-crustacyanin. Moving from *H. gammarus* to *H. americanus* for complex isolation, together with integrated biophysical characterization, resulted in crystals of β-crustacyanin that diffracted to 2.75 Å resolution and crystals of α-crustacyanin that diffracted to 6.3 Å resolution, together with high-quality negative-stain and preliminary cryo-EM images.

## 2. Materials and methods

### 2.1. *H. americanus* genome search

**Figure 2:**
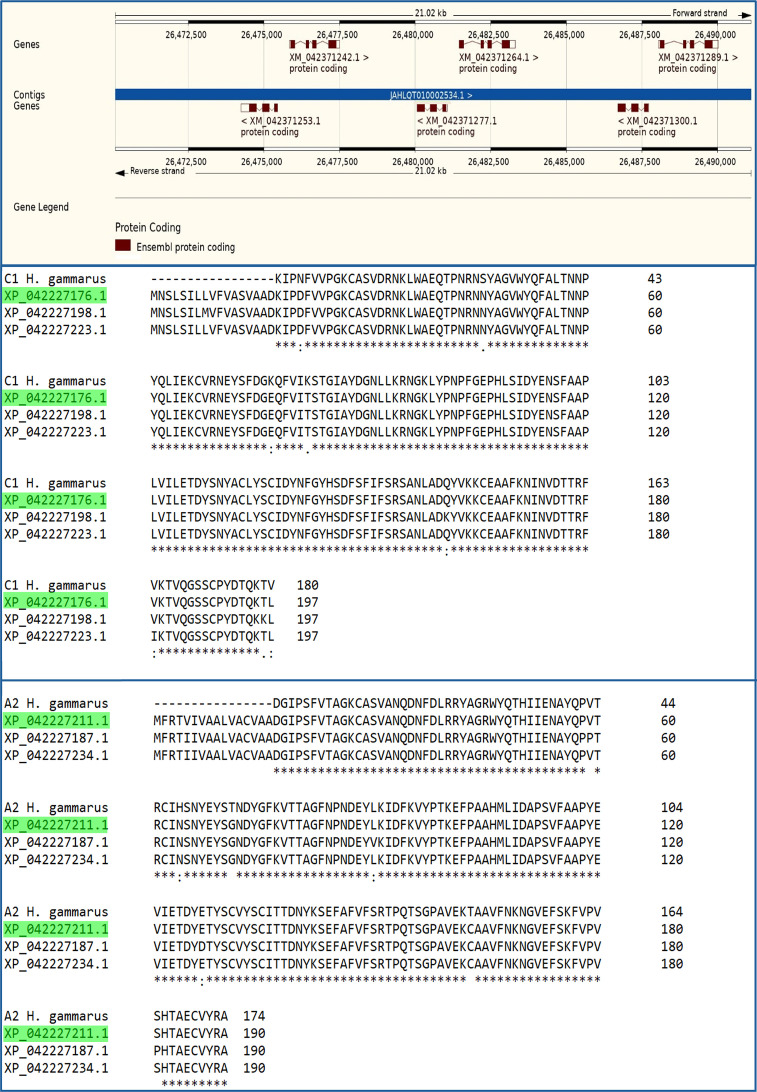
Top panel: gene location of the three genes (LOC121869686, LOC121869706 and LOC121869723) coding for three single proteins of type I [CRTC; XP_042227176.1 (XM_042371242.1), XP_042227198.1 (XM_042371264.1) and XP_042227223.1 (XM_042371289.1), respectively] and for the three genes (LOC121869696, LOC121869714 and LOC121869734) coding for three single proteins of type II [CRTA; XP_042227187.1 (XM_042371253.1), XP_042227211.1 (XM_042371277.1) and XP_042227234.1 (XM_042371300.1), respectively]. Multiple sequence alignment of type I (middle panel) and type II (bottom panel) proteins from *H. americanus* against those from *H. gammarus*. Amino acids conserved in all sequences are marked with a star, conserved amino acids are indicated with a colon and nonconserved amino acids are indicated with a dot. The FASTA sequences identified for the structures XP_042227176.1 with UniProt code A0A8J5NDD0 corresponding to CRTC subunit H1 and XP_042227211.1 with UniProt code A0A8J5NC20 corresponding to CRTA subunit H2 are highlighted in green.

The *H. americanus* genome (Polinski *et al.*, 2021 ▸) present in the NCBI database (Sayers *et al.*, 2025 ▸) was searched with the *BlastP* algorithm (Altschul *et al.*, 1997 ▸) using the sequence of the apo C1 subunit from *H. gammarus* (Gordon *et al.*, 2001 ▸) and the sequence of the apo A2 subunit from *H. gammarus* (Cianci *et al.*, 2002 ▸) as queries (March 2023) (Fig. 2 ▸). Sequence alignments were performed using the *MUSCLE* algorithm (Edgar, 2004 ▸).

### 2.2. Isolation and purification protocol for α- and β-crustacyanin

α-Crustacyanin and β-crustacyanin proteins were purified *ex vivo* from frozen tails of *H. americanus* by adapting previously available protocols for *H. gammarus* (Zagalsky, 1985 ▸). Frozen tails of *H. americanus* of certified origin were purchased from a local fishmonger (D.I.MAR. s.r.l., Via Don Giovanni Bosco 21/23, 60127 Ancona, Italy). All steps were conducted at 4°C under low-light conditions. Lobster carapace was quickly separated from the underlying hypodermis, reduced into small pieces and then spread over absorbing paper to dry overnight. The pieces were then ground into a powder using an electric coffee grinder. Grinding was conducted using short bursts to avoid overheating of the grinder denaturing the protein.

Powdered lobster shell (30 g) was sifted through a 25 mesh sieve and resuspended in 800 ml 300 m*M* Tris–borate buffer pH 6.8. The suspension was stirred overnight, ensuring that the pH remained below 8.5, and was then filtered through paper with mild suction. The dry cake obtained was washed twice with 300 m*M* Tris–borate buffer pH 6.8 and then with water, and was subsequently left to dry for 30 min with the suction pump kept on. The dry cake was transferred to a 2 l beaker containing 500 m*M* EDTA buffer pH 7.5 (1 g shell per 40 ml buffer). The beaker was shielded from light and left to stir overnight.

After overnight stirring, the suspension was filtered as above and the dry cake was washed once with EDTA. The blue-coloured filtrate was centrifuged at 3500 rev min^−1^ for 30 min in order to discard residual particles.

Ammonium sulfate was added to the clarified, blue-coloured solution to 50% saturation to precipitate the proteins by salting out. The pH value was maintained at 7.5 by adding 1 *M* HCl while stirring. The solution was left under stirring overnight and then centrifuged at 13 500 rev min^−1^ for 30 min. The resulting pellet was resuspended in 35 ml 200 m*M* monopotassium phosphate buffer (KH_2_PO_4_) pH 7.0 and dialyzed against 50 m*M* KH_2_PO_4_ buffer pH 7.0. The dialyzed solution was then fractionally precipitated to 30% ammonium sulfate saturation to remove unwanted proteins, and after centrifugation the supernatant was precipitated again to 50% ammonium sulfate saturation to finally isolate the proteins of interest by salting out.

After resuspension of the resulting pellet in 200 m*M* KH_2_PO_4_ buffer pH 7.0 (10 ml), the sample was dialyzed as described above.

**Figure 3:**
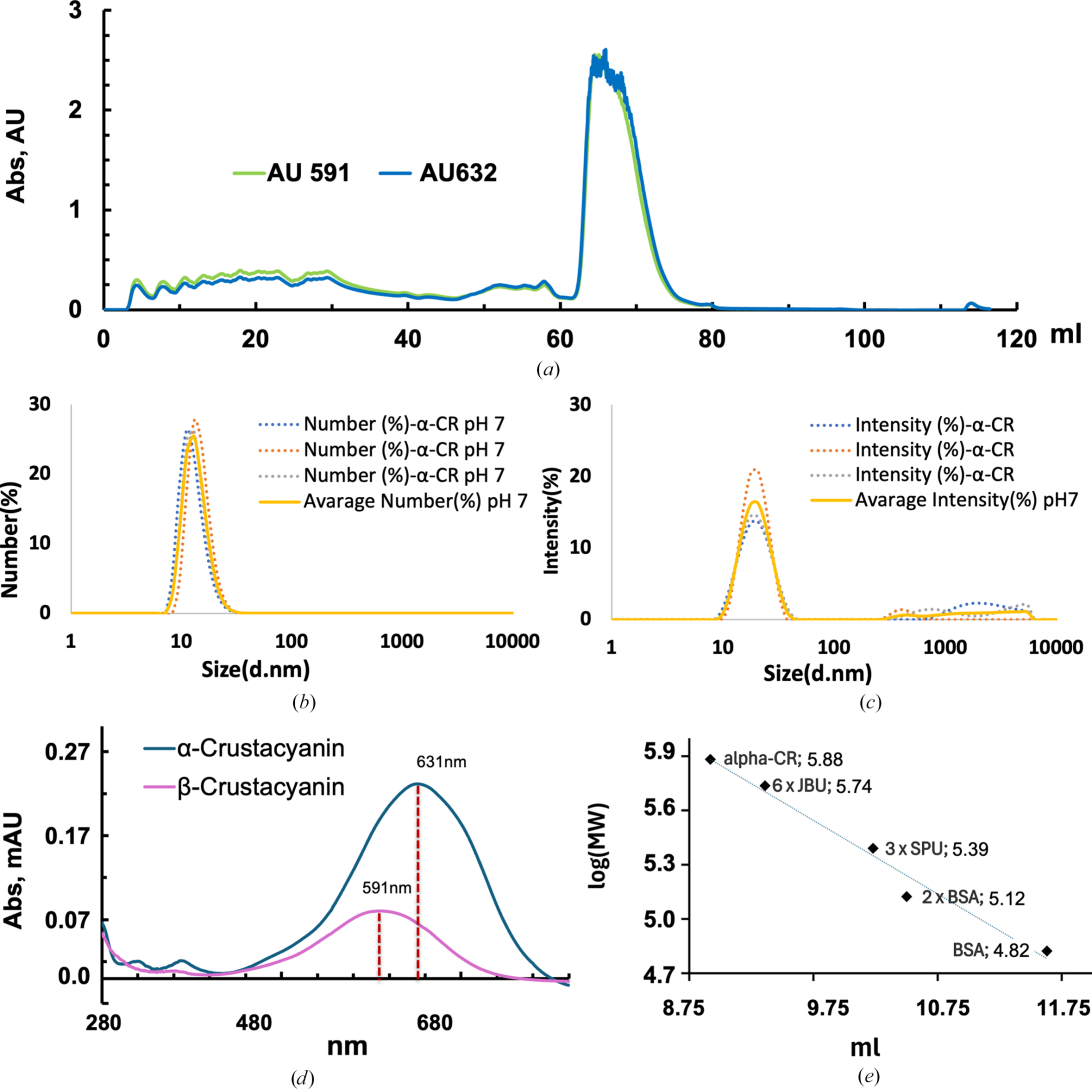
Purification steps and biochemical characterization of *H. americanus* α- and β-crustacyanins. (*a*) Ion-exchange chromatograms for α-crustacyanin and the loose heterodimeric β-crustacyanin subunits. (*b*, *c*) DLS analysis showing a homogenous population of monodisperse particles of α-crustacyanin. (*d*) Absorption spectra of purified β-crustacyanin (purple curve) and α-crustacyanin (blue curve) with maxima at 591 and 631 nm, respectively. (*e*) Sepharose 12 size-exclusion chromatography of α-crustacyanin with molecular-weight (MW) markers [6 × JBU (jack bean urease), MW = 544 482 kDa; 3 × SPU (*Sporosarcina pasteurii* urease), MW = 245 588 kDa; 2 × BSA (bovine serum albumin), MW = 132 864 kDa; BSA, MW = 66 432 kDa].

**Figure 4:**
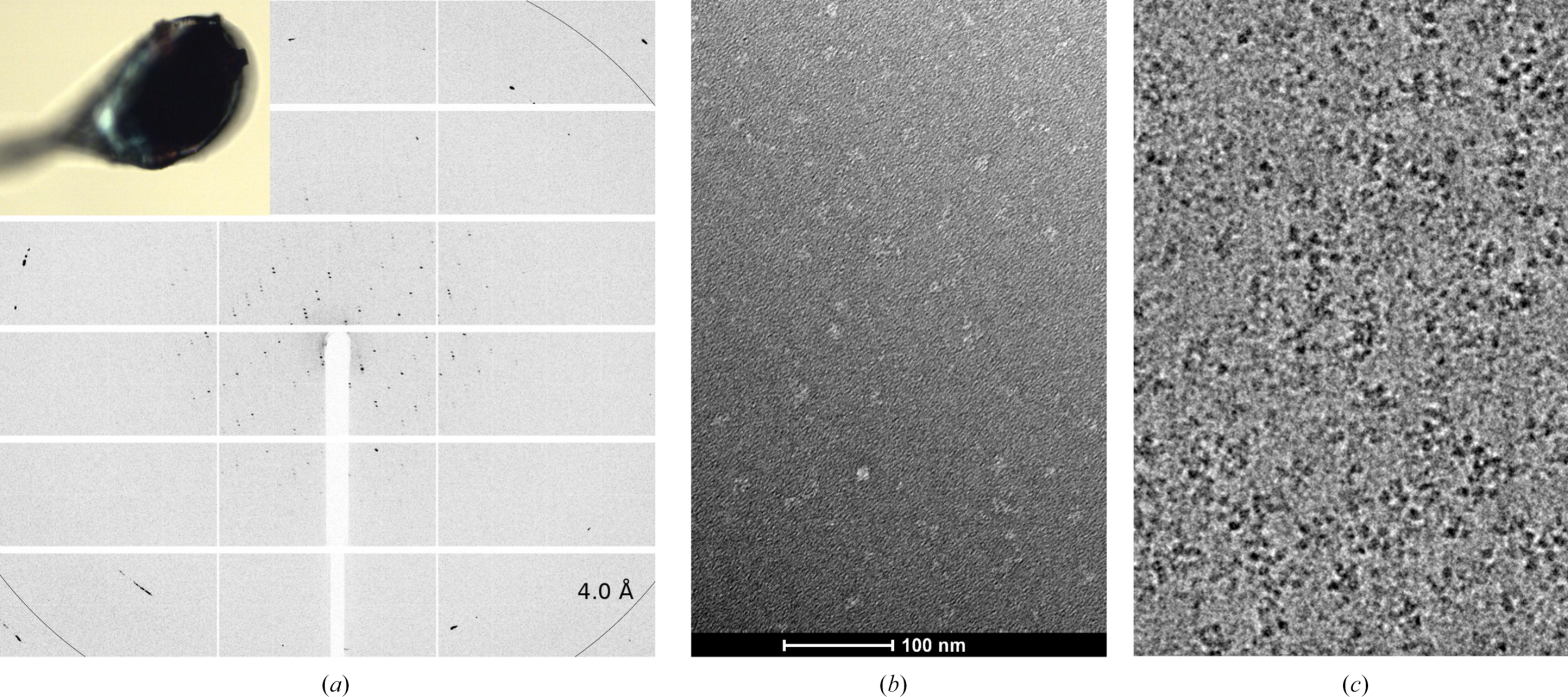
Preliminary diffraction and electron microscopy images from *ex vivo* purified α-crustacyanin. (*a*) Diffraction image extending to 6.3 Å resolution; the inset shows a mounted blue crystal on a nylon loop before data collection. (*b*) Representative micrograph of negative stain at a defocus of −1.5 µm. (*c*) Cryo-EM micrograph at a defocus of −1.2 µm. The largest particles are roughly 45 nm in length.

The sample was then loaded onto a TSK DEAE-5-PW column (5 ml) pre-equilibrated with 50 m*M* KH_2_PO_4_ buffer pH 7.0. After washing with the same buffer, elution was performed with a linear gradient of KCl from 0 to 1 *M* in the same buffer. Fractions were assayed for the presence of the different forms of crustacyanin (β and α) by reading the absorbance at 591 and 632 nm, respectively (Fig. 3 ▸*a*). Fractions containing α- and β-crustacyanin were pooled and concentrated by ultrafiltration through a 10 kDa membrane (Amicon Ultra centrifugal filters). The final preparations (8 mg ml^−1^ for α-crustacyanin and 7 mg ml^−1^ for β-crustacyanin) were stored at 4°C and used in subsequent analyses. The UV–Vis absorption spectra of purified β- and α-crustacyanin were recorded using a Shimadzu UV-1900i spectrophotometer in the range 200–800 nm (Fig. 4 ▸*d*). Samples were diluted in their elution buffer. Quartz cuvettes with a 1 cm path length were used. Spectra were baseline-corrected against the same buffer.

### 2.3. β-Crustacyanin crystallization and data collection

Vivid blue crystals of β-crustacyanin were obtained by the sitting-drop vapour-diffusion method in MRC 2-well crystallization plates by mixing 0.5 µl protein solution (7 mg ml^−1^ in 50 m*M* KH_2_PO_4_ buffer pH 7) with equal amounts of precipitant solution (100 m*M* MES pH 6.5, 1.6 *M* magnesium sulfate; condition E9, kit 206L; Jena Bioscence, Germany) without optimization. Crystals grew to a final size of about 90 µm within three weeks at a controlled temperature of 291 K (Table 1 ▸). Diffraction data were collected on beamline ID30B at the European Synchrotron Radiation Facility (ESRF), Grenoble, France (McCarthy *et al.*, 2018 ▸). The crystals were transported to the synchrotron in plates, mounted in nylon loops and flash-cooled directly at 100 K in a nitrogen gas stream without further cryoprotection. Diffraction data from two crystals were integrated with the *XDS* program package (Kabsch, 2010 ▸) within *autoPROC* (Vonrhein *et al.*, 2011 ▸) and scaled using *AIMLESS* (Evans, 2006 ▸). Data-collection statistics are summarized in Table 2 ▸.

### 2.4. Dynamic light scattering of α-crustacyanin

The proteins were assessed for monodispersity and stability prior to crystallization and cryo-EM experiments. Dynamic light scattering (DLS) was used to confirm the monodispersity of the sample in the elution buffer at pH 7 (Figs. 3 ▸*b* and 3 ▸*c*). α-Crustacyanin was used at 0.1 mg ml^−1^ in the elution buffer and each measurement was made in triplicate at 25°C. The pH was adjusted using 500 m*M* HCl in the case of acid pH and 500 m*M* NaOH in the case of basic pH. DLS experiments were carried out on a Malvern Zetasizer Pro instrument (Malvern Panalytical, UK) in back-scattering geometry (θ = 173°). The DLS results were analysed using the Zetasizer Advance *ZS Xplorer* software using the *CONTIN* method (v.3.00; https://www.malvernpanalytical.com/en/support/product-support/software/zetasizer-ultra-pro-zs-xplorer-software-update-v3-00), and the size distribution in number is considered here.

### 2.5. α-Crustacyanin crystallization and data collection

Dark blue crystals of α-crustacyanin were obtained by the sitting-drop vapour-diffusion method in MRC 2-well crystallization plates by mixing 1 µl protein solution (8 mg ml^−1^ in 50 m*M* KH_2_PO_4_, 200 m*M* KCl buffer pH 7) with an equal amount of precipitant solution (2.0 *M* ammonium dihydrogen phosphate pH 8.5, 100 m*M* Tris). Crystals grew to a final size of about 150 µm within three weeks at a controlled temperature of 291 K (Fig. 4 ▸*a*; Table 1 ▸).

Diffraction data were collected on beamline ID30B at the ESRF, Grenoble, France (McCarthy *et al.*, 2018 ▸). The crystals were transported to the synchrotron in plates, mounted in nylon loops and flash-cooled directly at 100 K in a nitrogen gas stream. Screening of dozens of crystals permitted the identification of a suitable crystal for data collection (Fig. 4 ▸*a*). The diffraction data were integrated with the *XDS* program package (Kabsch, 2010 ▸) within *autoPROC* (Vonrhein *et al.*, 2011 ▸) and were scaled using *AIMLESS* (Evans, 2006 ▸). Data-collection statistics are summarized in Table 2 ▸.

### 2.6. Grid preparation for negative-stain and cryo-EM data collection

Cryo-EM tests were performed at the Imaging Facility of the City University of New York Advanced Science Research Center, New York, USA. Grids were glow-discharged using a Fischione M1070 NanoClean to make their surface hydrophilic.

Preliminary negative-stain images were collected on a ThermoFisher Tecnai G2 Spirit Twin TEM to check for monospersity and optimize the sample concentrantion (0.06 mg ml^−1^ in 50 m*M* KH_2_PO_4_, 200 m*M* KCl buffer pH 7) on the grids (carbon film, 400 mesh copper, Electron Microscopy Sciences, USA) stained with uranyl acetate (Fig. 4 ▸*b*). For cryo-EM sample preparation, ∼4 µl of sample (0.6 mg ml^−1^, the same buffer as for negative staining) was deposited onto the grids (Quantifoil R1.2/1.3 Mesh 300, Germany) at 100% humidity and 10°C; the grids were then blotted with filter paper for 2 s using a Vitrobot Mark IV (ThermoFisher) and rapidly plunge-frozen in liquid ethane to allow the formation of a thin layer of amorphous ice with the particles embedded within it (Fig. 4 ▸*c*). Images were recorded using a ThermoFisher Titan Halo 80-300 TEM microscope equipped with X-FEG high-brightness gun at 300 kV and a Gatan K3 summit direct detection camera and were visualized using *cryoSPARC* v.3.2 (Punjani *et al.*, 2017 ▸).

## 3. Results and discussion

Searching the *H. americanus* genome (Polinski *et al.*, 2021 ▸) for the apo C1 subunit from *H. gammarus* (Gordon *et al.*, 2001 ▸), identified three genes (LOC121869686, LOC121869706 and LOC121869723) coding for three single proteins (type I; CRTC; XP_042227176.1, XP_042227198.1 and XP_042227223.1), each consisting of 197 amino acids with 96.7% sequence identity to the *H. gammarus* counterpart.

Similarly, searching for the sequence of the apo A2 subunit from *H. gammarus* (Cianci *et al.*, 2002 ▸) identified three genes (type II; CRTA; LOC121869696, LOC121869714 and LOC121869734) coding for three single proteins (XP_042227187.1, XP_042227211.1 and XP_042227234.1), each consisting of 190 amino acids (Fig. 2 ▸) with 98.3% sequence identity to the *H. gammarus* counterpart. Overall, the protein sequence alignment of subunits H1 and H2 from *H. americanus* versus C1 and A2 from *H. gammarus* reveals only a limited number of point mutations (Fig. 2 ▸). In H1 the mutations are N5D, S30N, K61E and K66T, while in H2 the mutations are H48N, T55G and T147C.

The high sequence identity between the proteins encoded by the three genes for both type I and type II subunits appears to confirm the presence of homogeneous pigments in *H. americanus*. These two triplets of genes are located among the gene families involved in cuticle formation and remodelling, which also encode chitinases (Polinski *et al.*, 2021 ▸), cuticular and chitin-binding proteins, and thus are possibly expressed at different levels during these stages.

The purified β-crustacyanin absorbed at λ_max_ = 591 nm, confirming the integrity and the nature of the complex (Fig. 3 ▸*d*). The sample yielded crystals that diffracted to 2.75 Å resolution (Table 2 ▸), with a net gain of 0.5 Å in resolution when compared with the homolog from *H. gammarus* (Cianci *et al.*, 2002 ▸). The criteria used for judging the resolution limits were 〈*I*/σ(*I*)〉 > 1.0 or 〈*I*〉 half-set correlation CC_1/2_ > 0.30 applied first. These two criteria have been extensively debated (Evans & Murshudov, 2013 ▸; Evans, 2006 ▸; Karplus & Diederichs, 2012 ▸, 2015 ▸). For β-crustacyanin data at 〈*I*/σ(*I*)〉 > 1.0, CC_1/2_ was still > 0.5. Using 〈*I*/σ(*I*)〉 > 1.5 or 〈*I*/σ(*I*)〉 > 2.0 as criteria the resolution limits would have been 2.96 and 3.04 Å, respectively, thus excluding a significant proportion of intensities above the noise level. The increase in the resolution of the crystallographic data is of particular interest, since the electron-density maps could reveal new details about the water network around the astaxanthin chromophores.

Previous crystallization conditions were obtained using microbatch under oil (Chayen *et al.*, 1990 ▸) and the resulting crystal structure presented a dodecane molecule, possibly arising from the paraffin oil, positioned between the two astaxanthins (Cianci *et al.*, 2002 ▸). The new crystallization conditions have been found with the vapour-diffusion method, and these could yield a crystal structure free from alkanes.

The integrity of *H. americanus* α-crustacyanin was assessed during purification by monitoring the absorption-spectrum peak at λ_max_ = 631 nm (Fig. 3 ▸*d*). Dynamic light-scattering (DLS) results confirmed the absence of protein aggregates in the sample, revealing a monodisperse particle population (Figs. 3 ▸*b* and 3 ▸*c*). Using this preparation, for the first time ever, it was possible to obtain diffracting crystals of α-crustacyanin, albeit only to a resolution of 6.3 Å, which could be indexed to obtain a space group and unit cell (Fig. 4 ▸*a*, Table 2 ▸). The criteria used for judging the resolution limits of the α-crustacyanin data were the same as used for the β-crustacyanin data. For α-crustacyanin data at 〈*I*/σ(*I*)〉 > 1.0, CC_1/2_ was 0.368. Using 〈*I*/σ(*I*)〉 > 1.5 or 〈*I*/σ(*I*)〉 > 2.0 as criteria, the resolution limits would have been 6.85 and 7.13 Å, respectively.

Taking the calculated molecular weight of a β-crustacyanin unit of 40.1 kDa as a reference, the estimated number of β-crustacyanin units in the asymmetric unit would range from 22, when considering a Matthews coefficient of 2.5 Å^3^ Da^−1^, to 11, when considering a Matthews coefficient of 5 Å^3^ Da^−1^, similar to that observed for β-crustacyanin (Cianci *et al.*, 2002 ▸). Gel filtration of the α-crustacyanin complex resulted in an approximate average molecular weight of around 762 kDa (Fig. 3 ▸*e*), which would correspond to a complex of 18 β-crustacyanin units.

Preliminary attempts to solve the crystal structure by molecular replacement using a single copy of the β-crustacyanin heterodimer have so far been unsuccessful. This led us to address structure solution using single-particle cryo-EM. Initial negative-stain data and preliminary cryo-EM images confirmed the monodispersity of the α-crustacyanin particles (Figs. 4 ▸*b* and 4 ▸*c*) and the suitability of the preparation for further data collection.

In conclusion, an integrated biophysical characterization of pigments from *H. americanus* resulted in the production, for the first time ever, of crystals of the α-crustacyanin complex, which diffracted to 6.3 Å resolution, and high-quality negative-stain and cryo-EM images. The resolution for β-crustacyanin also increased to 2.75 Å, promising novel insights into the binding of astaxanthin within the lipocalin calyx and a high-resolution model for fitting cryo-EM data.

Understanding the structure of the α-crustacyanin complex and its role in the colouration of the lobster carapace would provide a platform for the rational design of astaxanthin scaffolding compounds with tailored light-absorption properties for food pigments with enhanced nutraceutical properties or bio-inspired artificial light-harvesting mimetic systems.

## Figures and Tables

**Table 1:** Crystallization conditions for β-crustacyanin and α-crustacyanin

	β-Crustacyanin	α-Crustacyanin
Method	Vapour diffusion	Vapour diffusion
Plate type	Sitting drop	Sitting drop
Temperature (K)	291	291
Protein concentration (mg ml^−1^)	7	8
Buffer composition of protein solution	50 m*M* KH_2_PO_4_ pH 7	50 m*M* KH_2_PO_4_, 20 m*M* KCl pH 7
Composition of reservoir solution	100 m*M* MES pH 6.5, 1.6 *M* magnesium sulfate	2.0 *M* ammonium dihydrogen phosphate pH 8.5, 100 m*M* Tris
Volume of drop (µl)	0.5 + 0.5	1.0 + 1.0
Volume of reservoir (µl)	100	100
Time of growth (days)	21	15
Final crystal form and size (µm)	Rods, 90 × 30 × 30	Cuboids, 150 × 70 × 70

**Table 2:** Crystallographic data-collection statistics for β-crustacyanin and α-crustacyanin Values in parentheses are for the highest resolution shell.

	β-Crustacyanin	α-Crustacyanin
Beamline	ID30B, ESRF	ID30B, ESRF
Detector	EIGER	PILATUS 6M
Distance (mm)	301.8, 302.2	647.45
Rotation range (°)	0.05	0.1
Total range (°)	75 + 75	100
Wavelength (Å)	0.873	0.885
Temperature (K)	100	100
Space group	*P*6_3_22	*P*222_1_
*a*, *b*, *c* (Å)	121.6, 121.6, 184.9	123.7, 175.0, 403.4
α, β, γ (°)	90, 90, 120	90, 90, 90
Resolution range (Å)	184.9–2.75 (2.88–2.75)	132.2–6.32 (6.65–6.32)
Total reflections	508009 (69385)	68934 (16545)
Multiplicity	23.4 (24.6)	3.6 (3.7)
Completeness (%)	100.0 (100.0)	98.5 (98.5)
Mean *I*/σ(*I*)	8.0 (1.0)	6.4 (1.0)
*R*_merge_ †	0.406 (4.676)	0.082 (1.222)
*R*_p.i.m._ ‡	0.118 (1.341)	0.061 (0.895)
CC_1/2_	0.985 (0.52)	0.998 (0.368)

† *R*_merge_ = 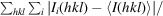

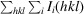
, where *I*_*i*_(*hkl*) is the intensity of a reflection and 〈*I*(*hkl*)〉 is the mean intensity of all symmetry-related reflections.

‡ *R*_p.i.m._ = 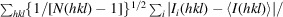

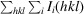
, where *I*_*i*_(*hkl*) is the intensity of a reflection, 〈*I*(*hkl*)〉 is the mean intensity of all symmetry-related reflections and *N* is the multiplicity.
